# Demographic/clinicopathological characteristics and prognosis of resectable Epstein-Barr virus-associated gastric cancer: a nested case-control study from an Eastern China

**DOI:** 10.3389/fonc.2025.1602091

**Published:** 2026-01-07

**Authors:** Lihu Gu, Qiufeng Zhang, Yuying Hu, Zhiyi Xiang, Shengqiang Ji, Weiming Yu, Xingchen Liu, Ping Chen, Feng Wu, Qi Zheng

**Affiliations:** 1 Department of General Surgery, Ningbo No. 2 Hospital, Ningbo, Zhejiang, China; 2 The First Affiliated Hospital of Zhejiang Chinese Medical University (Zhejiang Provincial Hospital of Chinese Medicine), Hangzhou, Zhejiang, China; 3 Department of General Surgery, The First Affiliated Hospital of Ningbo University, Ningbo, Zhejiang, China; 4 Ningbo Clinical Pathology Diagnosis Center Ningbo, Ningbo, Zhejiang, China; 5 Department of Gastrointestinal Surgery, The Affiliated Lihuili Hospital, Ningbo University, Ningbo, Zhejiang, China; 6 Intensive care Unit, Ningbo No. 2 Hospital, Ningbo, Zhejiang, China

**Keywords:** Epstein–Barr virus, gastric cancer, prognosis, CEA, pTNM

## Abstract

**Background:**

Epstein-Barr virus-associated gastric cancer (EBVaGC) represents a distinct molecular subtype of gastric cancer (GC). This multicenter study aimed to investigate the clinicopathological characteristics and prognosis of resectable EBVaGC patients.

**Methods:**

Data were prospectively collected and retrospectively analyzed from 1,400 patients at Ningbo No. 2 Hospital from January 2014 to December 2023 and 55 EBVaGC patients were identified. Additionally, 95 EBVaGC patients from two external cooperative centers were included. A 1:4 propensity score matching (PSM) analysis was performed between EBVaGC patients and EBV-negative gastric cancer (EBVnGC) patients. Median follow-up duration was 34 months.

**Results:**

Among the 150 EBVaGC patients, the median age was 64, and 88.7% were male. Undifferentiated tumors were more common. During the follow-up, 121 EBVaGC patients did not experience recurrence. After PSM, there were 137 EBVaGC patients and 548 EBVnGC patients. In EBVaGC patients, elevated Carcinoembryonic Antigen (CEA) levels (HR = 8.11, p=0.025) and pathological tumor-node-metastasis (pTNM) stage III (HR = 19.57, p=0.008) were independent risk factors for overall survival (OS). For disease-free survival (DFS), elevated CEA levels (HR = 6.23, p=0.035) and pTNM stage III (HR = 18.51, p=0.007) were independent risk factors. There was no significant difference in OS between the two groups (p=0.204). Compared to EBVnGC patients, EBVaGC patients showed a trend towards better DFS, although this did not reach statistical significance(p=0.061).

**Conclusion:**

EBVaGC patients exhibit unique clinicopathological characteristics and may show a trend toward better prognosis compared with EBVnGC patients, although this difference did not reach statistical significance.

## Introduction

Gastric cancer (GC) remains a major global health burden, ranking fifth in cancer incidence and mortality. Data from a study published in 2024 indicated over 968,000 new cases and nearly 660,000 fatalities from GC (1). In a seminal study conducted in 1993, Tokunaga and associates utilized *in situ* hybridization (ISH) technology to confirm the presence of Epstein-Barr virus-encoded small RNAs (EBERs) in GC cells (2). In 2014, The Cancer Genome Atlas (TCGA) classified GC into four distinct subtypes based on molecular profiles: microsatellite instability (MSI), genomic stability (GS), chromosomal instability (CIN), and EBV-associated gastric cancer (EBVaGC) (3).

EBVaGC is one of the predominant subgroups among EBV-associated tumors. The proportion of EBVaGC among all GC cases varies significantly across geographic regions, with reported rates ranging from 1.3% to 20.1% (4–7). This subtype is characterized by clonal expansion of EBV-infected cells. It exhibits distinct molecular features such as phosphatidylinositol-4,5-bisphosphate 3-kinase catalytic subunit alpha (PIK3CA) mutations, hypermethylation of DNA, and overexpression of programmed cell death ligand 1 (PD-L1) and programmed cell death ligand 2 (PD-L2), suggesting that EBV infection contributes to the malignant transformation of normal cells in gastric carcinogenesis (3, 8). Studies have revealed a higher incidence of EBVaGC in males than females, with a predilection for the proximal stomach or the proximal part of the residual stomach post-surgery (9). Notably, EBVaGC is associated with a relatively low rate of lymph node metastasis and a more favorable prognosis following surgical resection (10). Furthermore, patients with EBVaGC demonstrate a positive response to immune checkpoint inhibitors, highlighting its potential as a target for therapeutic interventions and a valuable biomarker (11).

While the unique aspects of EBVaGC are receiving increased recognition, there remains ongoing debate concerning its epidemiological features and prognostic implications. Geographical variations exist in the prevalence of EBVaGC across different regions (5, 12). More importantly, whether EBVaGC confers a more favorable prognosis compared to EBV-negative gastric cancer (EBVnGC) remains inconclusive, and the potential clinicopathological determinants influencing its prognosis warrant systematic elucidation (5, 6). It is worth noting that most research in this area is hampered by limitations such as small sample sizes and other potential sources of bias. To overcome these gaps​​, this case-control study collected data from patients with resectable GC across multiple Chinese medical institutions, aiming to thoroughly investigate the clinical characteristics of EBVaGC patients who underwent radical gastrectomy and to explore their prognosis.

## Methods

### Patients

The following inclusion criteria determined the eligibility of this study: 1) histopathologically confirmed primary gastric adenocarcinoma; 2) no history of other malignant tumors; 3) availability of comprehensive clinical and pathological data. Additionally, patients who underwent radical surgery were included in the final analysis. Based on these criteria, this study identified 1,400 GC patients who underwent surgical treatment at Ningbo No. 2 Hospital from January 2014 to December 2023, of which 55 were diagnosed with EBVaGC through ISH. Simultaneously, 95 EBVaGC patients diagnosed with ISH from the First Affiliated Hospital of Ningbo University and Ningbo Medical Center Li Huili Hospital were enrolled within the same time frame under identical criteria. Clinical data were prospectively collected and subsequently analyzed retrospectively. Specimen collection and utilization were approved by the Ethics Committee of Ningbo No. 2 Hospital (Ethics Approval Number: PJ-NBEY-KY-2019-153-01). The study was conducted according to the ethical standards of all participating institutions, and written informed consent was obtained from patients through signed consent forms.

### EBV ISH examination

**Figure 1:**
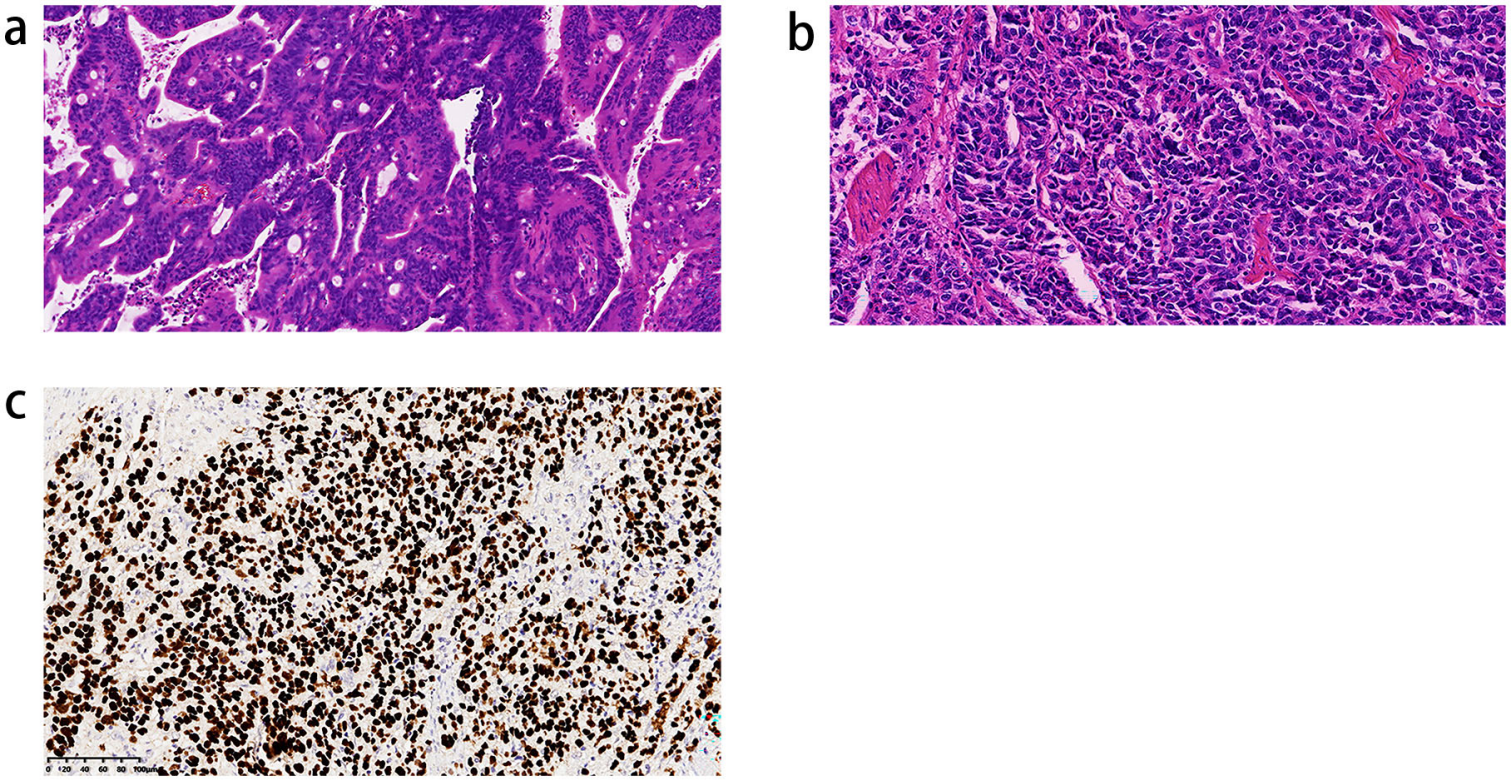
Pathological sections demonstrating differences between EBVnGC and EBVaGC patients (**(a)** EBVnGC group, **(b)** EBVaGC group, **(c)** EBER-ISH staining EBVaGC).

ISH is recognized as the gold standard for diagnosing EBVaGC in GC tissues. When tumor cells in gastric specimens treated by surgery show positive results for EBV-ISH, the diagnostic criteria for EBVaGC are designated as “EBV positive”. The EBV-ISH procedure uses EBER probes, detection kits, ISH protease, and staining solution. Formalin-fixed paraffin-embedded (FFPE) tissue sections were cut into 3 μm slices after dewaxing and pretreated with ISH protease. Hybridization and visualization were performed using fluorescently labeled probes and detection kits and restaining with a staining solution. For EBV-ISH, representative FFPE tissue sections that best reflected the overall histopathological characteristics of each GC case were selected. Three pathologists independently evaluated ISH results. **Figures 1a, b** show the pathological images of EBVnGC and EBVaGC. Representative staining of a case of EBVaGC by ISH is shown in **Figure 1c**, demonstrating specific nuclear positivity for EBER.

### Clinical and pathological data

Prospective clinical data related to patient characteristics were collected, including basic demographic data (age, sex, body mass index (BMI), comorbidities) and cancer-related information (tumor location, carcinoembryonic antigen (CEA) levels, type of gastrectomy, and postoperative treatment). Additionally, pathological features included degree of differentiation, tumor size, perineural invasion, lymphovascular invasion, pathological tumor-node-metastasis (pTNM) staging, number of lymph nodes dissected, and expression levels of human epidermal growth factor receptor 2 (HER-2) and mismatch repair proteins (MMR). Pathological staging was performed according to the American Joint Committee on Cancer (AJCC) Staging Classification for Gastric Cancer (8th edition, 2016).

### Follow-up

This study utilized information from outpatient and inpatient records, as well as physical examination, imaging, endoscopy, and laboratory test results obtained via telephone, text messages, and social media platforms, to systematically monitor the disease status of patients. In the first two years following surgery, follow-up intervals were set at every 3–6 months, followed by annual check-ups thereafter. Patients lost to follow-up were designated as censored patients, and the date of the last known contact was recorded. Overall survival (OS) was described as the time from the date of surgery to death from any cause. Disease-free survival (DFS) was defined as the duration from surgery to the first occurrence of local recurrence, distant metastasis. All patients participating in this study were followed up until December 2024.

### Statistical analysis

Statistical analysis was performed using SPSS 25.0. To reduce bias, propensity score matching (PSM) was performed between the EBVaGC and EBVnGC groups at a ratio of 1:4, with a caliper width of 0.02. Variables included in PSM were age, sex, BMI, tumor size, CEA level, perineural and lymphovascular invasion, pTNM stage, number of lymph nodes dissected, and postoperative chemotherapy. For normally distributed data, continuous variables were presented as mean ± standard deviation (SD), and for data that did not conform to a normal distribution, the median with interquartile range (IQR) was presented, with data comparison applying independent samples t-test or Wilcoxon rank-sum test. Categorical variables were expressed as frequency and percentage, and their data comparison was analyzed using Pearson’s chi-square test or Fisher’s exact test according to expected frequency. The Cox proportional hazards model was used for univariate and multivariate analysis of OS and DFS, with results presented as hazard ratio (HR) and 95% confidence interval (CI). Univariate analysis was conducted first for all potential prognostic factors. Variables with a P< 0.05 or those considered to have clinically relevant and potentially important associations with prognosis were then selected for multivariate analysis. Survival curves were plotted using the Kaplan-Meier method and compared using the log-rank test. All statistical tests were two-sided, with p < 0.05 considered statistically significant.

## Results

### Study population

**Figure 2:**
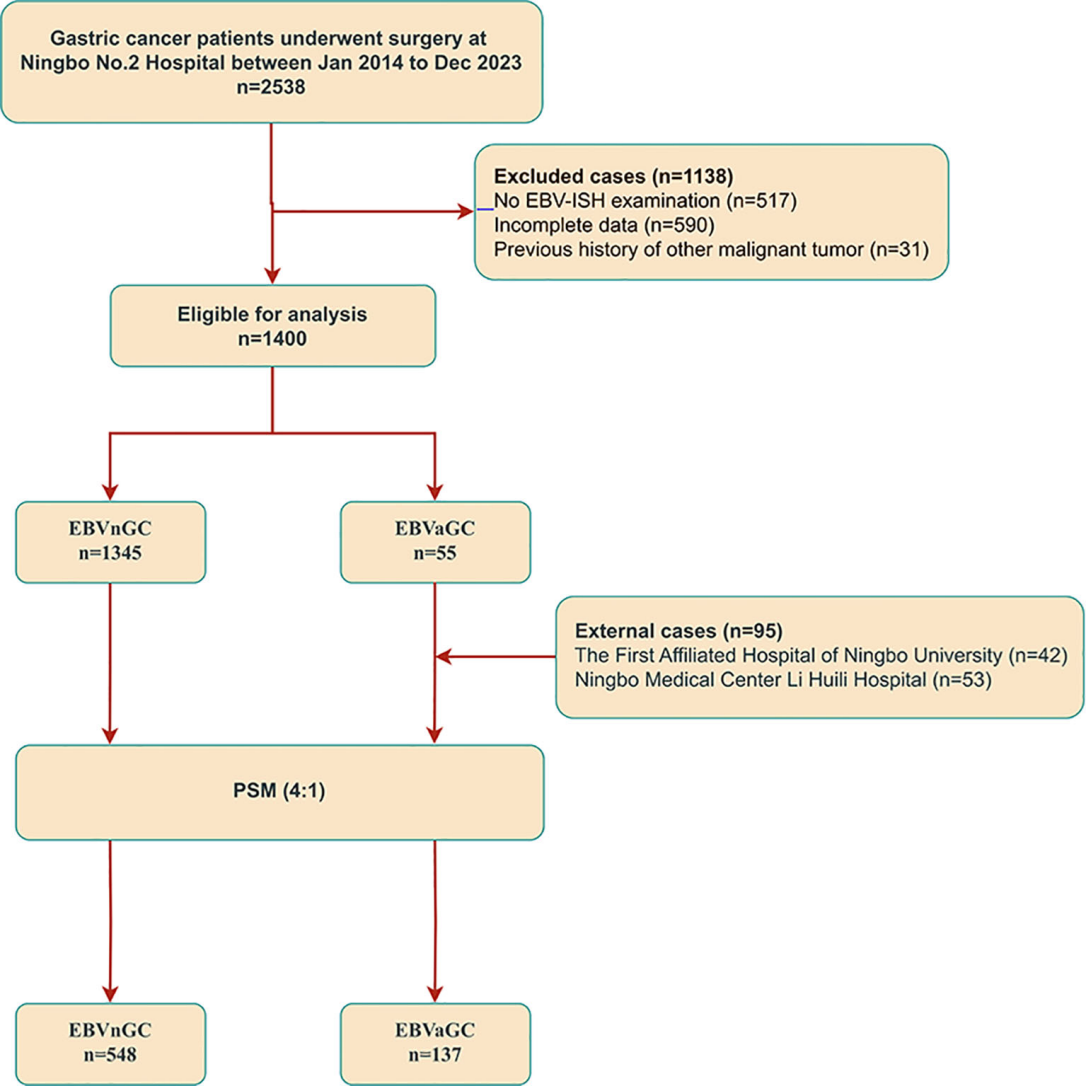
Flowchart detailing the selection process for the study participants.

From January 2014 to December 2023, a total of 2,538 patients underwent gastrectomy at Ningbo No. 2 Hospital. Based on the inclusion criteria, 1,138 patients were excluded, including 517 without EBV-ISH examination, 590 with incomplete data, and 31 with a history of other malignant tumors. Ultimately, 1,400 patients met the analysis criteria. Among them, 55 cases of EBVaGC were detected through ISH examination. In addition, this study included 42 patients from the First Affiliated Hospital of Ningbo University and 53 patients from Ningbo Medical Center Li Huili Hospital who were confirmed with EBVaGC through ISH. A total of 150 patients with EBVaGC from Eastern China were enrolled in this study. After excluding 13 patients who did not undergo radical surgery, a four-to-one match was conducted, including 685 patients, of which 548 were EBVnGC patients, and 137 were EBVaGC patients. For more details, see **Figure 2**.

### Clinical and pathological characteristics of EBVaGC patients

The baseline clinical characteristics of the 150 EBVaGC patients from Eastern China are shown in **Table 1**. The average age of the patients was 64 years, with an average BMI of 22.6. Male patients accounted for 88.7%, and female patients accounted for 11.3%. The 48.7% had comorbidities, including hypertension, coronary heart disease, diabetes, etc. Among all patients, 5.3% had a serum CEA level higher than 5 ng/mL. In terms of tumor location, 34% were located in the middle third of the stomach, 30% in the lower third, and 25% in the upper third. The most common type of gastrectomy was total gastrectomy (59.3%), followed by distal gastrectomy (36%) and proximal gastrectomy (2%). The 66.7% of patients received postoperative chemotherapy.

**Table 1:** Baseline clinical characteristics of patients with EBVaGC.

Clinical features	N=150
Age (years) (mean ± SD)	64.3 ± 9.1
Gender	
Male	133 (88.7%)
Female	17 (11.3%)
BMI (kg/m^2^) (mean ± SD)	22.6 ± 3.0
Comorbidity	
Absence	24 (16.0%)
Presence	73 (48.7%)
NA	53 (35.3%)
CEA (ng/mL)	
≤5	93 (62.0%)
>5	8 (5.3%)
NA	49 (32.7%)
Tumor location	
Upper third	44 (25.0%)
Middle third	51 (34.0%)
Lower third	45 (30.0%)
At least two-thirds	10 (6.7%)
Surgical type	
Proximal stomach	3 (2.0%)
Distal stomach	54 (36.0%)
Remnant stomach	4 (2.7%)
Total stomach	89 (59.3%)
Postoperative chemotherapy	
Absence	50 (33.3%)
Presence	100 (66.7%)

EBVaGC, EBV-associated gastric cancer; SD, standard deviation; BMI, body mass index; CEA, carcinoembryonic antigen; NA, not available.

**Table 2** describes the pathological characteristics of the 150 EBVaGC patients from Eastern China in detail. Undifferentiated tumors accounted for 66%, and differentiated tumors accounted for 34%. In terms of HER-2 expression, most patients were HER-2 negative (83.3%), and only 2% were HER-2 positive. Patients with mismatch repair-proficient (pMMR) accounted for 82.7%, those with mismatch repair-deficient (dMMR) accounted for 1.3%, and the MMR status of the remaining 16% was not clear. Patients with a maximum tumor diameter of ≤5 cm accounted for 73.3%. Perineural invasion was present in 49.3%, and lymphatic invasion in 54.7%. In the pTNM, stage I accounted for 34%, stage II for 23.3%, and stage III for 42.7%. The median number of lymph nodes dissected was 25.5. The median follow-up time in the queue was 34 months.

**Table 2:** Pathological characteristics of EBVaGC patients.

	N=150
Differentiation	
Differentiated	51 (34.0%)
Undifferentiated	99 (66.0%)
Tumor size (cm)	
≤5	110 (73.3%)
>5	40 (26.7%)
Perineural invasion	
Absence	76 (50.7%)
Presence	74 (49.3%)
Lymphovascular invasion	
Absence	68 (45.3%)
Presence	82 (54.7%)
pT category	
T1	32 (21.3%)
T2	27 (18.0%)
T3	30 (20.0%)
T4a	55 (36.7%)
T4b	6 (4.0%)
pN category	
N0	71 (47.3%)
N1	28 (18.7%)
N2	20 (13.3%)
N3a	27 (18.0%)
N3b	4 (2.7%)
pTNM	
1a	29 (19.3%)
1b	22 (14.7%)
2a	21 (14.0%)
2b	14 (9.3%)
3a	33 (22.0%)
3b	24 (16.0%)
3c	7 (4.7%)
HER-2	
Negative	125 (83.3%)
Positive	3 (2.0%)
2+	22 (14.7%)
MMR	
pMMR	124 (82.7%)
dMMR	2 (1.3%)
NA	24 (16.0%)
Number of lymph node dissection (median, IQR)	26 (20-33)

EBVaGC, EBV-associated gastric cancer; pT, pathologic tumor staging; pN, pathologic node staging; pTNM, pathologic tumor, node and metastasis staging; HER-2, human epidermalgrowth factor receptor 2; MMR, mismatch repair; pMMR, mismatch repair-proficient; dMMR, mismatch repair-deficient; IQR, interquartile range.

### Prognosis of EBVaGC patients

**Table 3** describes the prognosis of the 150 EBVaGC patients from Eastern China. In terms of postoperative recurrence, by the end of the follow-up period, most patients (80.7%) did not experience recurrence, 15.3% had recurrence, and the recurrence data for the remaining patients were missing. During the observation period, 80.7% of the patients remained alive.

**Table 3:** Prognosis of patients with EBVaGC.

Prognostic parameters	N=150
Postoperative recurrence	
Absence	121 (80.7%)
Presence	23 (15.3%)
NA	6 (4.9%)
Number of patients surviving	121 (80.7%)
Follow-up period (median, IQR)	33.82 (13-42)

EBVaGC, EBV-associated gastric cancer; NA, not available; IQR, interquartile range.

### Factors affecting the prognosis of EBVaGC patients

**Supplementary Table 1** shows the results of Cox univariate analysis for OS in 137 EBVaGC patients, indicating that tumor location, lymphovascular invasion, and pTNM stage were potentially associated with OS. **Supplementary Table 2** shows the results of Cox univariate analysis for DFS in 137 EBVaGC patients, indicating that tumor location, tumor size, lymphovascular invasion, and pTNM stage were potentially associated with DFS. In **Table 4**, further Cox multivariate analysis revealed that elevated CEA levels and pTNM stage III were independent risk factors for OS and DFS in EBVaGC patients. Although age showed a certain risk trend, it did not reach statistical significance.

**Table 4:** Multivariate analysis to determine the risks of OS and DFS in 137 patients with EBVaGC.

Clinicopathological features	OS			DFS		
	HR	95%CI	P	HR	95%CI	P
Age (years)						
≤60	1			1		
>60	4.02	0.83-19.55	0.085	3.28	0.74-14.61	0.120
CEA (ng/mL)						
≤5	1			1		
>5	8.11	1.30-50.44	0.025	6.23	1.14-33.99	0.035
pTNM						
I/II	1			1		
III	19.57	2.18-175.42	0.008	18.51	2.19-156.77	0.007

OS, overall survival; DFS, disease-free survival; EBVaGC, EBV-associated gastric cancer; HR, hazard ratios; CI, confidence interval; CEA, carcinoembryonic antigen.

### PSM analysis

Through 4-to-1 PSM, no significant differences were found between the EBVnGC group (548 patients) and the EBVaGC group (137 patients) in terms of age, gender, BMI, tumor size, perineural invasion, lymphatic invasion, pTNM category, number of lymph nodes dissected, and postoperative chemotherapy (**Table 5**). There were significant differences in the types of surgeries received between the two groups of patients. Among the EBVnGC patients, 67.3% underwent distal gastrectomy, and 31.4% underwent total gastrectomy. In contrast, among the EBVaGC patients, 38.7% underwent distal gastrectomy, and 56.2% underwent total gastrectomy. More than half of the EBVnGC patients had differentiated tumors, whereas only 33.6% of the EBVaGC patients had differentiated tumors. Additionally, there were significant differences in the HER-2 status between the two groups. Among the EBVnGC patients, 74.5% were HER-2 negative, and 8.6% (47 out of 548) were HER-2 positive. In comparison, among the EBVaGC patients, 84.7% were HER-2 negative and 2.2% were HER-2 positive.

**Table 5:** Baseline characteristics of EBVnGC patients and EBVaGC patients after propensity score matching (4:1).

Clinicopathological feature	EBVnGC patients N=548	EBVaGC patients N=137	P
Age (years)			
≤60	188 (34.3%)	49 (35.8%)	
>60	360 (65.7%)	88 (64.2%)	0.748
Gender			
Male	490 (89.4%)	122 (89.1%)	
Female	58 (10.6%)	15 (10.9%)	0.901
BMI (kg/m^2^)			
<18.5	42 (7.7%)	10 (7.3%)	
18.5-23.9	329 (60.0%)	87 (63.5%)	
≥24	177 (32.3%)	40 (29.2%)	0.752
Comorbidity			
Absence	17 (3.1%)	23 (16.8%)	
Presence	295 (53.8%)	68 (49.6%)	
NA	236 (43.1%)	46 (33.6%)	<0.001
Surgical type			
Proximal stomach	7 (1.3%)	3 (2.2%)	
Distal stomach	369 (67.3%)	53 (38.7%)	
Remnant stomach	0 (0)	4 (2.9%)	
Total stomach	172 (31.4%)	77 (56.2%)	<0.001
Tumor location			
Upper third	85 (15.5%)	36 (26.3%)	
Middle third	124 (22.6%)	47 (34.3%)	
Lower third	328 (59.9%)	44 (32.1%)	
At least two-thirds	11 (2.0%)	10 (7.3%)	<0.001
Tumor size (cm)			
≤5	408 (74.5%)	99 (72.3%)	
>5	140 (25.5%)	38 (27.7%)	0.601
Differentiation			
Differentiated	297 (54.2%)	46 (33.6%)	
Undifferentiated	251 (45.8%)	91 (66.4%)	<0.001
Perineural invasion			
Absence	295 (53.8%)	69 (50.4%)	
Presence	253 (46.2%)	68 (49.6%)	0.467
Lymphovascular invasion			
Absence	257 (46.9%)	63 (46.0%)	
Presence	291 (53.1%)	74 (54.0%)	0.848
pT category			
T1	147 (26.8%)	30 (21.9%)	
T2	107 (19.5%)	26 (19.0%)	
T3	103 (18.8%)	25 (18.2%)	
T4a	166 (30.3%)	50 (36.5%)	
T4b	25 (4.6%)	6 (4.4%)	0.661
pN category			
N0	290 (52.9%)	66 (48.2%)	
N1	91 (16.6%)	26 (19.0%)	
N2	69 (12.6%)	17 (12.4%)	
N3a	83 (15.1%)	24 (17.5%)	
N3b	15 (2.7%)	4 (2.9%)	0.870
pTNM			
1a	126 (23.0%)	27 (19.7%)	
1b	87 (15.9%)	21 (15.3%)	
2a	76 (13.9%)	19 (13.9%)	
2b	62 (11.3%)	13 (9.5%)	
3a	94 (17.2%)	29 (21.2%)	
3b	82 (15.0%)	21 (15.3%)	
3c	21 (3.8%)	7 (5.1%)	0.889
Number of lymph node dissection (median, IQR)	24 (19-31)	25 (20-33)	0.227
HER-2			
Negative	408 (74.5%)	116 (84.7%)	
Positive	47 (8.6%)	3 (2.2%)	
2+	93 (17.0%)	18 (13.1%)	0.013
CEA (ng/mL)			
≤5	450 (82.1%)	87 (63.5%)	
>5	70 (12.8%)	8 (5.8%)	
NA	28 (5.1%)	42 (30.7%)	0.175
Postoperative chemotherapy			
Absence	217 (39.6%)	48 (35.0%)	
Presence	331 (60.4%)	89 (65.0%)	0.327

EBVaGC, EBV-associated gastric cancer; EBVnGC, EBV-negative gastric cancer; BMI, body mass index; pT, pathologic tumor staging; pN, pathologic node staging; pTNM, pathologic tumor, node and metastasis staging; IQR, interquartile range; IQR, interquartile range; HER-2, human epidermalgrowth factor receptor 2; CEA, carcinoembryonic antigen; NA, not available.

**Figure 3:**
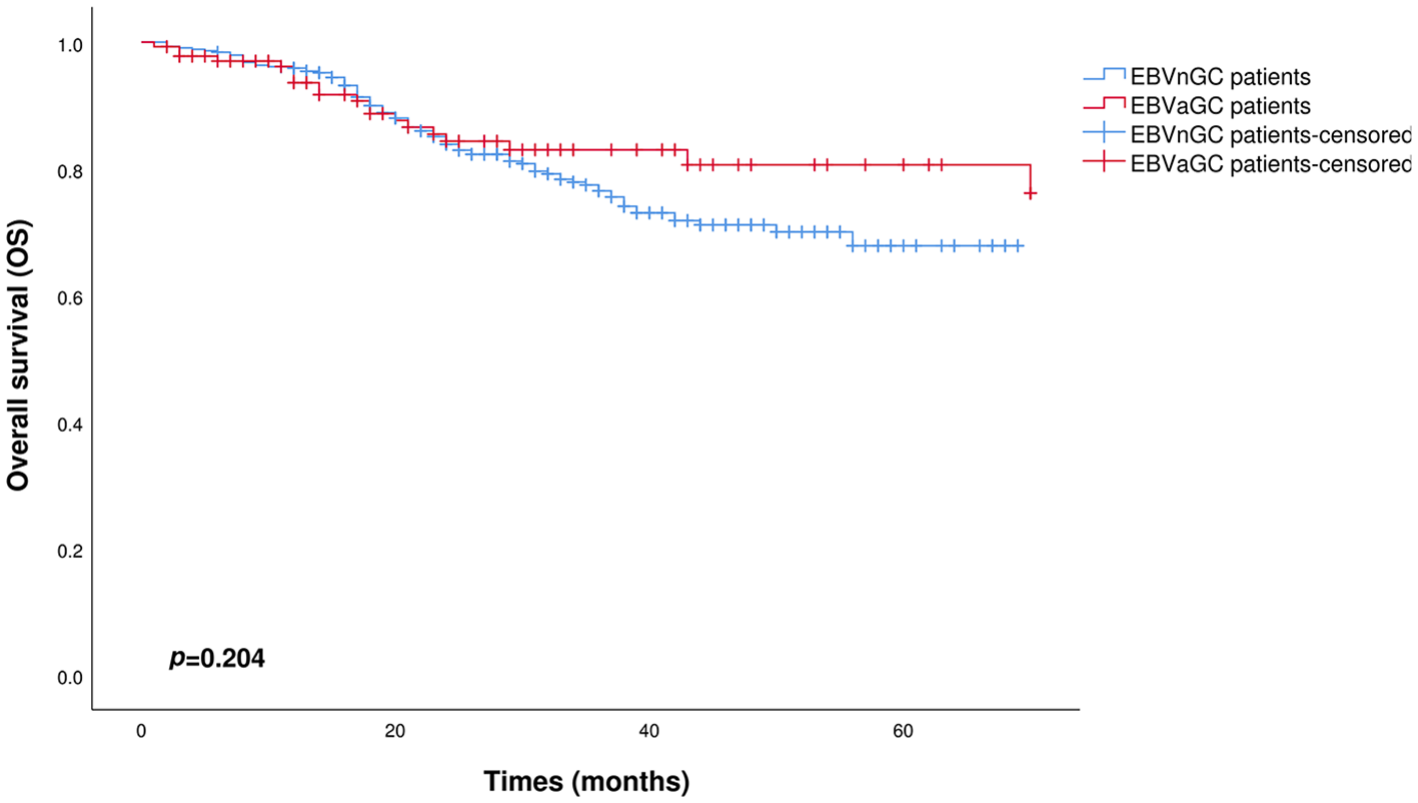
Kaplan-Meier survival curves comparing OS between EBVaGC and EBVnGC patients after PSM (p=0.204).

**Figure 4:**
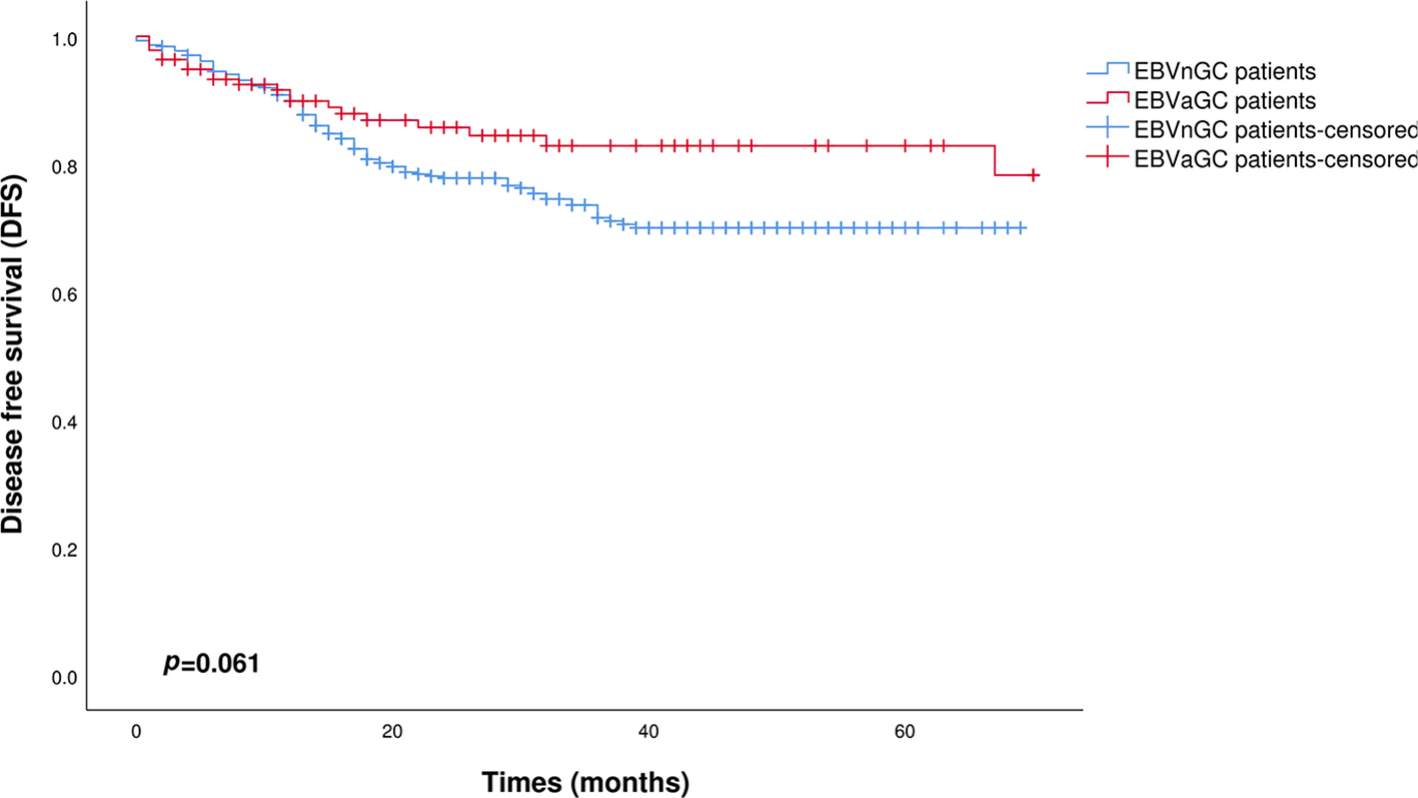
Kaplan-Meier survival curves comparing DFS between EBVaGC and EBVnGC patients after PSM (p=0.061).

**Figures 3** and **4** show the Kaplan–Meier curves for OS and DFS, respectively, between the EBVnGC group and the EBVaGC group after PSM processing. In this study, there was no significant difference in OS between the two groups (p=0.204). The DFS was better in the EBVaGC group compared to the EBVnGC group, but the difference did not reach statistical significance (p=0.061).

In the long-term prognosis of gastric cancer, both OS and DFS are influenced by various factors. The results show that advanced age, higher pTNM stage, and elevated CEA levels are independent risk factors, while EBV infection acts as a protective factor. For more details, see **Table 6**.

**Table 6:** Multivariate analysis to determine the risks of OS and DFS in 685 GC patients after propensity score matching (4:1).

Clinicopathological features	OS			DFS		
	HR	95%CI	P	HR	95%CI	P
Age (years)						
≤60	1			1		
>60	1.95	1.22-3.13	0.006	1.57	1.03-2.39	0.037
EBV-ISH						
negative	1			1		
positive	0.56	0.29-1.09	0.085	0.49	0.26-0.93	0.028
CEA (ng/mL)						
≤5	1			1		
>5	1.64	1.03-2.61	0.039	1.65	1.07-2.56	0.025
Perineural invasion						
Absence	1					
Presence	1.68	1.01-2.77	0.044	1.56	0.99-2.48	0.058
pTNM						
I	1			1		
II	2.51	1.01-6.27	0.048	3.52	1.39-8.95	0.008
III	6.34	2.67-15.06	<0.001	9.35	3.87-22.59	<0.001

OS, overall survival; DFS, disease-free survival; GC, gastric cancer; HR, hazard ratios; CI, confidence interval; EBV-ISH, Epstein-Barr encoding region *in situ* hybridization; CEA, carcinoembryonic antigen; pTNM, pathologic tumor, node and metastasis staging.

## Discussion

This study conducted a detailed analysis of the clinicopathological characteristics and prognosis of patients with resectable EBVaGC. For patients with EBVaGC who underwent radical surgery, a survival comparison was made with patients with EBVnGC after propensity score matching (PSM). The results showed that EBVaGC accounts for approximately 4% of all gastric adenocarcinoma. The average age of EBVaGC patients was 64. Among 150 patients with EBVaGC, 88.7% were male, reflecting a male predominance within this subgroup. These characteristics are similar to those reported in other regions of China and in international studies (4, 13, 14). As shown in **Table 5**, among the PSM-matched gastric cancer patients, a similar gender distribution was observed between patients with EBVnGC and EBVaGC, and no significant difference was found between the two groups. This may be due to the higher likelihood of male being exposed to risk factors such as smoking, alcohol consumption, and high-salt diets. A study from Japan showed that salt intake and occupational exposure may damage the gastric mucosa, increasing the risk of EBV infection (15). Additionally, gender differences in sex hormones and immune regulation may be factors. One research suggested that male sex hormones, such as testosterone, may suppress immune responses, while estrogen provides stronger immune control against EBV (16). Most EBVaGC patients were HER-2 negative. This suggests that EBV infection may inhibit differentiation and interfere with HER-2 expression through specific molecular pathways such as Latent Membrane Protein 1/2A (LMP1/2A)-mediated oncogenic signaling (3).

In the EBVaGC patients from Eastern China discussed in this study, tumors were predominantly located in the middle stomach. This distribution is likely attributable to the combined effects of EBV infection mechanisms, local immune responses, chronic inflammatory microenvironments, and molecular epigenetic features. The mucosa of the gastric body and cardia may recruit a large number of nonspecific lymphocytes through cytokines in the context of chronic inflammation, creating an immunosuppressive microenvironment conducive to EBV infection and tumor development (17). Meanwhile, EBV-driven DNA hypermethylation may affect the differentiation regulation of stem cells in the gastric body and cardia, leading to malignant transformation (18). Moreover, BamHI-A rightward transcript (BART) miRNAs encoded by EBV can promote tumor cell survival by inhibiting host tumor suppressor genes, and specific cell types in the middle stomach may be more sensitive to these miRNAs (17). Histologically, EBVaGC can be divided into three subtypes and lymphoepithelioma-like carcinoma (LELC) is more common. LELC often manifests as undifferentiated carcinoma with dense lymphocytic infiltration, resembling nasopharyngeal carcinoma (NPC) (19). This finding was confirmed in our study. Additionally, more than 80% of LELC cases have been reported to be EBV-positive (20).

It is generally believed that EBVaGC is associated with a better prognosis. In this study, the postoperative recurrence rate was 15.3%. Cox regression analysis showed that pTNM stage III is an independent risk factor for OS and DFS in EBVaGC patients. This indicates that the stage of pTNM has an important prognostic value for patients. CEA is an acidic glycoprotein with embryonic antigen characteristics that can enhance tumor invasiveness through its adhesion, immune suppression, and protease inhibition functions. Li et al. found that serum CEA levels in EBVaGC patients are lower than normal values, but no correlation was found between CEA levels and long-term prognosis (21). Further analysis in our study revealed that elevated CEA levels were an independent risk factor for OS and DFS in EBVaGC patients, suggesting that CEA may serve as a prognostic biomarker. After PSM analysis, no significant difference in OS was found between the EBVaGC and EBVnGC groups, but the DFS was better in the EBVaGC group. This result may be influenced by the limited sample size, insufficient follow-up duration, and other confounding factors.

The exact mechanisms by which EBV affects the prognosis of EBVaGC patients remain unclear, but it is widely acknowledged that its protective effects are closely related to immune factors. EBVaGC patients have a unique immune microenvironment, with more immune-active cells infiltrating the tumor microenvironment, such as CD8+ cytotoxic T cells and mature dendritic cells, forming an immune-activated phenotype (22, 23). This active immune environment may enhance immune surveillance and inhibit tumor progression, thereby providing a certain protective effect. Accordingly, the antitumor immune response is also enhanced in EBVaGC patients. Studies found a higher proportion of tumor-infiltrating lymphocytes (TILs) and significant increases in CD8+ T cells and cytotoxic T lymphocytes (CTLs) infiltration in these patients (24–26). These cells may inhibit tumor progression by directly killing tumor cells or modulating immune responses. Additionally, virus antigen-driven immune recognition may also play an important role. Viral proteins expressed during EBV latency, such as EBNA1 and LMP2A, can act as tumor-specific antigens to activate T cells and natural killer (NK) cells, forming continuous immune surveillance (27, 28). EBV infection also promotes an inflammatory response in GC tissues, which is closely related to interferon-γ-mediated signaling pathways and adaptive immune responses (29). Inflammatory responses are generally considered an important component of antitumor immunity, and thus, EBV infection may also indirectly inhibit tumor progression through this mechanism.

EBVaGC patients also exhibit unique genetic and molecular expression profiles that may inhibit tumor malignancy and reduce distant metastasis through epigenetic modifications and signaling pathway regulation. A significant feature of EBVaGC is genome-wide hypermethylation, which can lead to the silencing of tumor suppressor genes but may also suppress the expression of pro-metastatic genes (3, 30). For example, the promoter regions of genes such as PIK3CA and cyclin-dependent kinase inhibitor 2A (CDKN2A) show high levels of methylation in EBVaGC, which not only affects the expression of these genes but may also reduce metastatic potential by inhibiting tumor stemness. Studies found that high-frequency mutations in PIK3CA and activation of the Wnt pathway in EBVaGC promote clonal expansion, but hypermethylation may limit the overly invasive phenotype (31, 32). One research indicated that EBV infection induces fat mass and obesity-associated gene (FTO) expression through myelocytomatosis oncogene (MYC), inhibiting the translation of m6A-modified metastasis-related genes, thereby reducing lymph node metastasis (33).

The unique immune microenvironment and molecular expression profile of EBVaGC provide distinct advantages and potential for immunotherapy. PD-L1/PD-L2, important markers of tumor immune evasion, are highly expressed in EBVaGC, making them ideal targets for immune checkpoint inhibitor (ICI) therapy. Studies have shown that patients with EBVaGC have a higher overall response rate (ORR) to immunotherapy, with prolonged DFS and OS (34, 35). The special immune microenvironment of EBVaGC provides a “hot tumor” basis for immunotherapy, characterized by significant lymphocyte infiltration and an immune-activated phenotype (23). Specific immunotherapy strategies, such as EBV-specific CD8+ cytotoxic T-cell adoptive therapy, have shown success in nasopharyngeal carcinoma and lymphoma and may further optimize EBVaGC treatment by targeting EBV antigens in the future. In addition, chimeric antigen receptor T-cell (CAR-T) immunotherapy and cancer vaccines have also shown potential applications in the treatment of EBVaGC (36).

This study provides insights into the prognosis of EBVaGC patients from Eastern China through its large sample size and multicenter design. However, limitations such as the retrospective nature of the study, short follow-up duration and lack of data on immunotherapy restrict the comprehensiveness and applicability of the findings.

## Conclusion

This research found that EBVaGC patients have a better prognosis compared to those with EBVnGC. EBVaGC exhibits unique clinicopathological features, including a male predominance, a high rate of undifferentiation, a low HER-2 positivity rate, and significant lymphocytic infiltration. Additionally, elevated CEA levels and pTNM stage III classification were identified as independent risk factors for OS and DFS in EBVaGC patients.

## Data Availability

The raw data supporting the conclusions of this article will be made available by the authors, without undue reservation.

## Glossary

GC: Gastric Cancer
ISH: In Situ Hybridization
EBERs: Epstein-Barr virus-encoded small RNAs
TCGA: the Cancer Genome Atlas
MSI: microsatellite instability
GS: Genomic Stability
CIN: Chromosomal Instability
EBVaGC: Epstein-Barr Virus-associated gastric cancer
EBVnGC: Epstein-Barr Virus-negative Gastric Cancer
FFPE: Formalin-fixed paraffin-embedded;PSM, Propensity-Matched Analysis
OS: Overall Survival
CEA: Carcinoembryonic Antigen
DFS: Disease-Free Survival
BMI: Body Mass Index
pTNM: Pathological tumor-node-metastasis
HER-2: Human Epidermal Growth Factor Receptor 2
MMR: Mismatch Repair
AJCC: American Joint Committee on Cancer
SD: Standard Deviation
IQR: Interquartile Range
HR: Hazard Ratio
CI: Confidence Interval
PD-1: Programmed Cell Death ligand 1
PD-2: Programmed Cell Death ligand 2
PIK3CA: Phosphatidylinositol-4, 5-bisphosphate 3-kinase catalytic subunit alpha
pMMR: Patients with mismatch repair-proficient
dMMR: mismatch repair-deficient;BART, BamHI-A Rightward Transcript
LELC: lymphoepithelioma-like carcinoma
NPC: nasopharyngeal carcinoma
LMP1/2A: Latent Membrane Protein 1/2A
TILs: Tumor-infiltrating lymphocytes;CTLs, Cytotoxic T lymphocytes;CDKN2A, Cyclin-Dependent Kinase Inhibitor 2A
FTO: Fat mass and obesity-associated gene
MYC: Myelocytomatosis oncogene
ICI: Immune Checkpoint Inhibitor
ORR: Overall Response Rate
CAR-T: Chimeric Antigen Receptor T-cell
NK cells: Natural KillerCells;
