# Supplementary material for: Demographic/clinicopathological characteristics and prognosis of resectable Epstein-Barr virus-associated gastric cancer: a nested case-control study from an Eastern China
Source: Front Oncol. 2026 Jan 7;15:1602091. doi: 10.3389/fonc.2025.1602091 (PMC12819199; doi:10.3389/fonc.2025.1602091)
Supplement: Supplementary file 1 [file Table1.docx]

Supplementary Table 1. Univariate analysis to determine the risk of OS in 137 patients with EBVaGC.

|  | HR | 95%CI | P |
| --- | --- | --- | --- |
| Age (years) | | | |
| ≤60 | 1 |  |  |
| >60 | 1.74 | 0.66-4.60 | 0.261 |
| Gender | | | |
| Male | 1 |  |  |
| Female | 0.42 | 0.06-3.11 | 0.393 |
| CEA (ng/mL) | | | |
| ≤5 | 1 |  |  |
| >5 | 3.08 | 0.66-14.40 | 0.152 |
| Tumor location | | | |
| Upper third | 1 |  |  |
| Middle third | 0.17 | 0.02-1.48 | 0.108 |
| Lower third | 1.37 | 0.45-4.12 | 0.578 |
| At least two-thirds | 4.23 | 1.22-14.70 | 0.023 |
| Tumor size (cm) | | | |
| ≤5 | 1 |  |  |
| >5 | 2.077 | 0.86-5.02 | 0.104 |
| Perineural invasion | | | |
| Absence | 1 |  |  |
| Presence | 1.70 | 0.68-4.28 | 0.256 |
| Lymphovascular invasion | | | |
| Absence | 1 |  |  |
| Presence | 4.54 | 1.33-15.51 | 0.016 |
| pTNM | | | |
| Ⅰ | 1 |  |  |
| Ⅱ | 3.75 | 0.39-36.06 | 0.253 |
| Ⅲ | 11.77 | 1.56-88.76 | 0.017 |
| Postoperative chemotherapy | | | |
| Absence | 1 |  |  |
| Presence | 1.37 | 0.50-3.76 | 0.547 |

OS, overall survival; EBVaGC, EBV-associated gastric cancer; HR, hazard ratios; CI, confidence interval; CEA, carcinoembryonic antigen; HER-2, human epidermalgrowth factor receptor 2; pTNM, pathologic tumor, node and metastasis staging.
