# Supplementary material for: Demographic/clinicopathological characteristics and prognosis of resectable Epstein-Barr virus-associated gastric cancer: a nested case-control study from an Eastern China
Source: Front Oncol. 2026 Jan 7;15:1602091. doi: 10.3389/fonc.2025.1602091 (PMC12819199; doi:10.3389/fonc.2025.1602091)
Supplement: Supplementary file 2 [file Table2.docx]

Supplementary Table 2. Univariate analysis to determine the risk of DFS in 137 patients with EBVaGC.

|  | HR | 95%CI | P |
| --- | --- | --- | --- |
| Age (years) | | | |
| ≤60 | 1 |  |  |
| >60 | 1.66 | 0.63-4.38 | 0.306 |
| Gender | | | |
| Male | 1 |  |  |
| Female | 0.41 | 0.06-3.10 | 0.390 |
| CEA (ng/mL) | | | |
| ≤5 | 1 |  |  |
| >5 | 3.02 | 0.65-8.56 | 0.160 |
| Tumor location | | | |
| Upper third | 1 |  |  |
| Middle third | 0.16 | 0.19-1.36 | 0.093 |
| Lower third | 1.40 | 0.47-4.22 | 0.546 |
| At least two-thirds | 4.23 | 1.22-14.67 | 0.023 |
| Tumor size (cm) | | | |
| ≤5 | 1 |  |  |
| >5 | 2.66 | 1.11-6.39 | 0.029 |
| Perineural invasion | | | |
| Absence | 1 |  |  |
| Presence | 1.81 | 0.72-4.55 | 0.205 |
| Lymphovascular invasion | | | |
| Absence | 1 |  |  |
| Presence | 4.79 | 1.40-16.36 | 0.012 |
| pTNM | | | |
| Ⅰ | 1 |  |  |
| Ⅱ | 4.07 | 0.42-39.16 | 0.224 |
| Ⅲ | 13.16 | 1.74-99.28 | 0.012 |
| Postoperative chemotherapy | | | |
| Absence | 1 |  |  |
| Presence | 1.48 | 0.54-4.08 | 0.448 |

DFS, disease-free survival; EBVaGC, EBV-associated gastric cancer; HR, hazard ratios; CI, confidence interval; CEA, carcinoembryonic antigen; HER-2, human epidermal growth factor receptor 2; pTNM, pathologic tumor, node and metastasis staging.
